# Differentiated Effects of Allyl Isothiocyanate in Diabetic Rats: From Toxic to Beneficial Action

**DOI:** 10.3390/toxins14010003

**Published:** 2021-12-21

**Authors:** Monika Okulicz, Iwona Hertig, Tomasz Szkudelski

**Affiliations:** Department of Animal Physiology, Biochemistry and Biostructure, Faculty of Veterinary Medicine and Animal Science, Poznan University of Life Sciences, Wołyńska 35, 60-637 Poznan, Poland; iwona.hertig@up.poznan.pl (I.H.); tomasz.szkudelski@up.poznan.pl (T.S.)

**Keywords:** allyl isothiocyanate, diabetes, metabolism, rats

## Abstract

Allyl isothiocyanate (AITC), a constituent of Brassica family plants, has been reported to possess a high bioactivity in animal and human cells, showing ambiguous properties from adverse to beneficial ones. It was reported its genotoxic, carcinogenic, goitrogenic effects. On the other side, AITC has shown anti-cancer, cardioprotective, neuroprotective, and lately anti-obesity abilities. So far, its anti-diabetic effects are poorly explored. We tried to assess AITC action on carbohydrate, lipid and hormonal disorders in high fat diet-fed/streptozotocin diabetic rats. In this report, diabetic rats were treated intragastrically at doses 2.5, 5 and 25 mg/kg b.w./day of AITC for 2 weeks. Irrespectively of doses, AITC considerably lowered thyroid hormones (fT4, fT3), increased liver TG content, and also caused robust LDL-cholesterol and direct bilirubin concentration enhancement. Moreover, AITC at the highest dose caused pancreatic amylase and lipase drops and thyroid gland hypertrophy. AITC at 2.5 and 5 mg significantly reduced blood glucose levels along with robust beta-hydroxybutyric acid drop. Additionally, AITC at 5 mg improved insulin sensitivity (HOMA-IR index) in spite of reduced blood insulin. To conclude, despite amelioration of diabetic hyperglycemia by AITC, the adverse lipids and hormonal effects may exclude its use as a health-promoting compound in terms of anti-diabetic properties.

## 1. Introduction

Allyl isothiocyanate (AITC) is one of the most wide-spread isothiocyanates in the plant kingdom that are used for food and condiments. This compound is formed by myrosinase hydrolysis of the glucosinolate sinigrin. AITC has shown both adverse as well as beneficial impact on health status of humans and animals. The clastogenicity, genotoxicity, mutagenicity of AITC has been observed in bacterial and mammalian cells in vitro and in vivo. Reactive oxygen species might be involved in the genotoxic effects of AITC [1,2]. Moreover, increased thyroid mass and decreased iodine uptake were observed in studies conducted to investigate the goitrogenic effects of AITC in rats [3]. On the other side, AITC has been considered as a nutrition supplement for its preventive and medicinal effect on some types of cancer [4,5,6] and it was recognized as a potent antioxidant [7,8]. AITC exhibited cytotoxic effects on cancer cells by inducing cell cycle arrest, apoptosis, inhibition of cell metastasis [9]. AITC showed also the immunomodulatory effects, such as enhancement of total white blood cells [10] as well as bone marrow cellularity [11]. AITC positive effects seems to occur at or near to its cytotoxic dose. Recently, the great interest has been to find the capacity of biactive compounds to prevent and treat obesity and its comorbidities such as type 2 diabetes. Diabetes is a metabolic disease-causing carbohydrate, lipid and hormonal homeostasis disorders. Hiperglycemia and dyslipidemia in type 2 diabetes disturb functionality mainly of such organs and tissues such as pancreas, liver, kidney, skeletal muscles and fat tissue. Lately, the in vivo study of Yamada-Kato et al. [12] demonstrated that wasabi leaf which, contains very high amounts of AITC, suppressed obesity in rats fed a high-fat diet (HFD) due to the upregulation of expression of β_3_ adrenergic receptors in brown adipose tissue. When mice were given AITC in addition to HFD, they were protected from body weight gains, organ hypertrophy, dyslipidemia and hepatic steatosis through the modulation of mitochondrial activity [13]. AITC was reported to reduce hiperglycemia during intraperitoneal glucose tolerance test [14]. Very recently, Sahin and co-researchers [15] indicated that 100 mg/kg b.w. AITC decreased the blood glucose concentrations by the improvement in GLUT2 expression in liver and kidney of diabetic rats. Further, it was reported that AITC metabolites may exert anti-obesity effects through suppression of adipogenesis or lipogenesis. Glutathione conjugate of AITC (GSH-AITC) and *N*-acetyl-*S*-(*N*-allylthiocarbamoyl)cysteine (NAC-AITC) effectively inhibit adipogenic differentiation of 3T3-L1 preadipocytes and suppress expression of PPARγ, which is up-regulated during adipogenesis [16]. AITC and NAC-AITC also improved pancreatic function by suppressing PPARγ phosphorylation caused by oxidative stress through the Nrf2 pathway [17]. All these results suggest that AITC prevents obesity and insulin resistance by inhibition of preadipocytes differentiation, inhibition of lipogenesis, activation of lipolysis and positive modulation of mitochondria activity as well as improving glucose transport.

In spite of these data, the anti-diabetic effects of AITC are poorly elucidated. In the present study, we assessed AITC influence on carbohydrate, lipid and hormonal disorders in HFD/streptozotocin-induced diabetic rats. AITC was applied at different doses to determine its potential toxic effects in diabetic animals.

## 2. Results

### 2.1. Effect of High-Fat Diet/STZ Injection and AITC on Carbohydrate and Lipid Parameters

**Figure 1:**
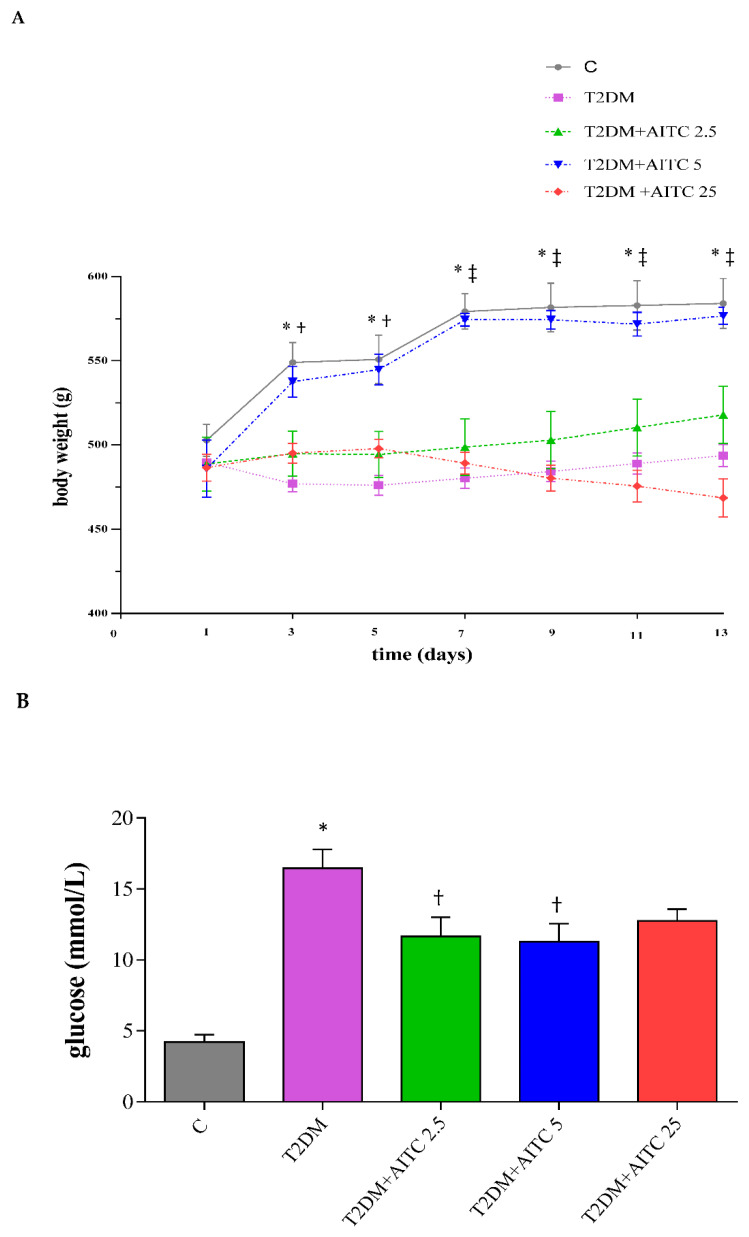

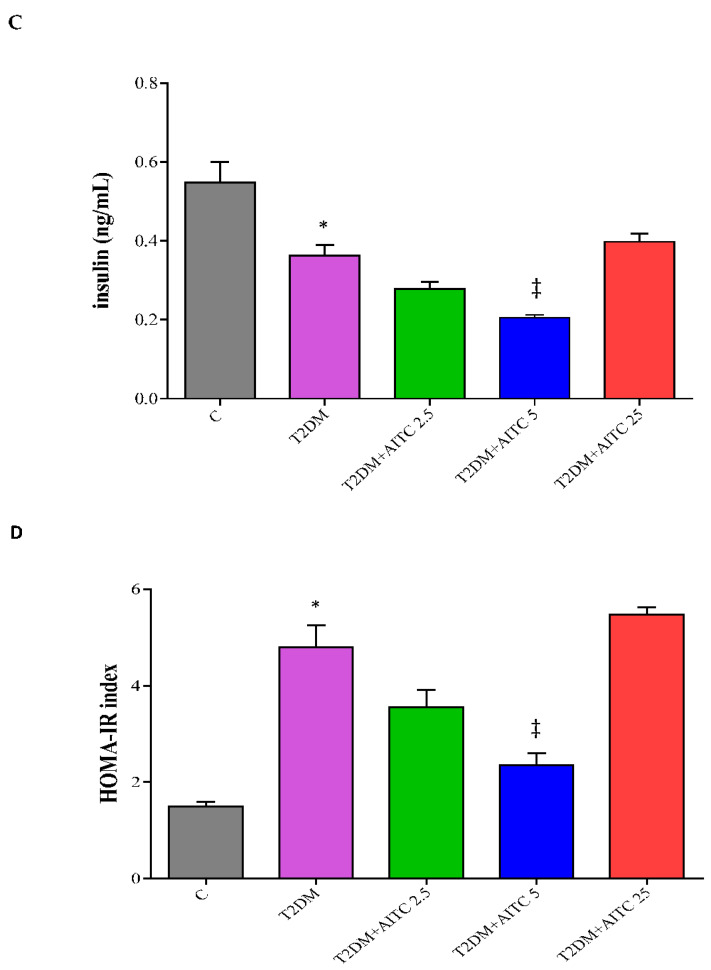
(**A**) Body weight gains of rats during experimental period (two-daily basis); Data are the mean ± SEM (*n* = 8). * *p* ≤ 0.01—significant difference between C and T2DM; † *p* ≤ 0.05—significant difference between T2DM and T2DM + AITC 5; ‡ *p* ≤ 0.01—significant difference between T2DM and T2DM + AITC 5. (**B**) The effect of AITC on glucose concentration (**C**) on insulin concentration (**D**) on insulin resistance (HOMA–IR index) in T2DM rats. (**B**–**D**) Each column represents the mean ± SEM (*n* = 8). * *p* ≤ 0.01 compared with C group; † *p* ≤ 0.05 compared with T2DM group; ‡ *p* ≤ 0.01 compared with T2DM group. Abbreviations: C—control group; T2DM–high-fat diet with STZ injected group; T2DM + AITC 2.5; AITC 5; AITC 25—high–fat diet with STZ injected group with oral administration of 2.5; 5; 25 mg/kg b.w. AITC, respectively.

In the T2DM group, we noted changes characteristic to type 2 diabetes such as significant increase in basal plasma glucose (*p* ≤ 0.01; Figure 1B), triglycerides (*p* ≤ 0.01; Table 1), insulin resistance (HOMA-IR index) (*p* ≤ 0.01; Figure 1D), decreased insulin concentration (*p* ≤ 0.01; Figure 1C) and glucose-dependent insulin-releasing polypeptide (GIP) drop (*p* ≤ 0.05; Table 2). Surprisingly, a significant liver glycogen increase as well as drop of LDL-cholesterol was noted compared with healthy rats (*p* ≤ 0.01; *p* ≤ 0.05; Table 1).

AITC administrated to diabetic rats was able to reduce glucose level at two applied doses 2.5 and 5 mg/kg b.w. (*p* ≤ 0.05; Figure 1B). However, only AITC at 5mg/kg b.w. ameliorated insulin resistance (*p* ≤ 0.01; Figure 1D). Independently on doses, AITC considerably deteriorated lipid homeostasis by increasing liver TG (*p* ≤ 0.01) and LDL-cholesterol concentrations (*p* ≤ 0.01). Additionally, AITC 2.5 mg/kg b.w. elevated TG in the serum (*p* ≤ 0.05) and the highest dose (25 mg/kg b.w.) caused considerable increase in FFA (*p* ≤ 0.01). Only 2.5 mg/kg b.w. of AITC increased cholesterol HDL (*p* ≤ 0.05) and 5 mg/kg b.w. decreased liver cholesterol (*p* ≤ 0.01) Table 1.

### 2.2. Effect of High-Fat Diet/STZ Injection and AITC on Thyroid, Pancreatic and Incretin Hormones

In the T2DM group we observed decrease in fT4 (*p* ≤ 0.01), compared with non-diabetic rats. Surprisingly, AITC at 5 mg/kg b.w. significant lowered insulin (*p* ≤ 0.01; Figure 1C), glucagon (*p* ≤ 0.01) and fT4 concentration in the serum (*p* ≤ 0.01). AITC at 2.5 mg/kg b.w. and 25 mg/kg b.w. decreased fT3 (*p* ≤ 0.05 and *p* ≤ 0.01; respectively). Moreover, the highest dose of AITC caused considerable GLP-1 drop (*p* ≤ 0.01) Table 2.

### 2.3. Effect of High-Fat Diet/STZ Injection and AITC on Toxicity Biomarkers

In the T2DM group the symptom of kidney nephropathy and pancreas failure was not observed. Even serum creatinine was lowered (*p* ≤ 0.01). However, the significant influence on liver was noted: lowered De Ritis ratio (*p* ≤ 0.01), AspAT level activity (*p* ≤ 0.05), urea level (*p* ≤ 0.05) and robust increase in direct bilirubin, beta-hydroxybutyric acid concentrations (*p* ≤ 0.01) as well as AlAT (*p* ≤ 0.05) and alkaline phosphatase activity (*p* ≤ 0.01).

AITC independently on doses bolstered an increase in direct bilirubin concentrations (*p* ≤ 0.01) and it caused a significant drop of beta-hydroxybutyric acid (*p* ≤ 0.01) as well as AlAT (*p* ≤ 0.05) compared with T2DM rats. Additionally, AITC 5 mg/kg b.w. decreased creatinine (*p* ≤ 0.05) and pancreatic amylase activity (*p* ≤ 0.05). What is interesting, AITC 25 mg/kg b.w. strongly exacerbated the pancreas status: pancreatic amylase (*p* ≤ 0.01) and lipase drop (*p* ≤ 0.05) as well as the liver status: decreased activity of alkaline phosphatase (*p* ≤ 0.01) Table 3.

### 2.4. Effect of High-Fat Diet/STZ Injection and AITC on Organ Mass and Body Weight Gains

AITC 25 mg/kg b.w. caused a significant increase relative mass of thyroid gland (*p* ≤ 0.05). However, relative liver mass was unchanged (Table 3). The body weight gains were significantly lower in T2DM group in comparison to C. AITC 5mg/kg b.w. considerably reversed the body mass gains during the experimental period in comparison to T2DM group (Figure 1A).

## 3. Discussion

In this study, we aimed at the evaluation of the AITC anti-diabetic potential in high-fat diet-fed/STZ diabetic rats. Lipid disorders are a very common feature of diabetes, mainly due to increased cholesterol and triacylglycerol as well as lower HDL cholesterol levels. AITC was previously shown to significantly prevent HFD-induced disregulation of lipid metabolism-related genes in the liver (decreased expression of markers hepatic lipogenesis-like fatty acids synthase; FAS) and sterol regulatory element binding protein (SREBP) [18]. Kim et al. [16] reported that, especially allyl isothiocyanate metabolites, such as GSH-AITC and NAC-AITC, might play an active role in the prevention of fatty liver development. AITC per se was also observed to decrease HFD-induced hepatic steatosis [13]. Contrary to this, Muztar et al. [19] shown high plasma phospholipid concentrations, increased total cholesterol, phospholipid and total lipid contents in the liver following AITC feeding to healthy rats. In our previous studies, 2.5 and 5 mg AITC also elevated cholesterol, but lowered triacylglycerols in the blood serum after 2 weeks administration to healthy rats [20]. This apparent inconsistency was also observed in the present study in diabetic conditions. Contrary to what was expected, AITC at 2.5 mg elevated TG levels in the serum and at all doses increased LDL-cholesterol. These effects are detrimental since worsen diabetic disturbances. On the other hand, we observed dose-dependent HDL-cholesterol increase and a decrease in liver cholesterol content in response to AITC treatment. The observed lipid metabolism abnormalities seemed to be, at least partially, the result of AITC antithyroid activity shown in the present study (tendency to thyroid weight increase and a significant decrease in serum levels of fT4 and fT3). The AITC antithyroid activity was observed first by Langer and Greer [3]. The relative thyroid mass was increased in healthy male Wistar rats administered 2 to 5 mg of AITC by gavage daily for 60 days. Moreover, Muztar et al. [19] confirmed binding of AITC with T4 in the in vitro study. It could explain a significant fT4 and fT3 drop observed at 5 mg and at 2.5 mg, 5mg AITC, respectively. According to Liberopoulos and Elisaf [21], overt or subclinical decrease in thyroid hormones levels were correlated with changes in lipoprotein concentrations, such as elevation of LDL-cholesterol, whereas HDL-cholesterol concentration was usually normal or even increased. The lipid profile, shown in the present study in rats treated with AITC, is consistent with these observations. These changes seemed to be associated with the thyroid hormone effects on the activities of key lipoprotein metabolism enzymes. Noted elevated HDL-cholesterol level at AITC 2.5 mg could be attributed to decreased cholesteryl ester transfer protein (CETP) activity. It is also highly probable that the observed hypertriglyceridemia was a results of reduced lipoprotein lipase (LPL) activity as a result of insulin decrease in our study (LPL is responsible for the triglyceride-rich lipoprotein hydrolysis and is activated by insulin). When blood levels of fT4 are low, decreased cholesterol content is frequently observed in the liver as a result of down-regulating 3-hydroxy-3-methylglutaryl-CoA reductase (HMG-CoA reductase) activity [21]. This was observed at AITC 5 mg. Unfortunately, all AITC doses significantly increased liver TG content. There are only three potential sources of the hepatic TG storage pool; de novo lipogenesis, plasma non-esterified fatty acids from adipose tissue and remnant lipoproteins [22]. Our study has shown the lack of significant FFA changes (except for the highest dose of AITC) and suggested a limited lipoprotein lipase activation due to insulin drops. Therefore, the most likely source of the hepatic TG storage pool was de novo lipogenesis activation in rats treated with AITC. A distinct effect of isothiocyanates per se on lipid metabolism was noted in our previous studies related to benzyl isothiocyanate (BITC) [23]. We have shown that the highest dose of AITC increased blood FFA levels in diabetic rats. This effect is unfavorable because is associated with insulin resistance, which develops into type 2 diabetes and indicates that supplementation of 25 mg AITC to diabetic rats impairs insulin action. Irrespective on thyroid hormone-related metabolic changes, it should be highlighted that the highest dose of AITC evoked an increase in thyroid mass with a concomitant decrease in blood fT3 levels. Given that fT3 is the most active thyroid hormone, both effects are adverse and indicate goitrogenic properties of 25 mg AITC.

Recently, the hepatoprotective activity of 5 and 50 mg AITC (oral administered once daily for 3 days) against carbon tetrachloride-induced liver injury in Sprague Dawley rats was shown [24]. AITC significantly reduced the ALAT activity level. However, our study indicates that AITC was not able to improve liver status in diabetic rats. Moreover, AITC considerably increased the bile flow obstruction and/or hepatocyte damage. Diabetes, as a disorder, leads not only to liver complications but also to renal failure. Renal dysfunction caused by AITC was reported by Lewerenz et al. [25]. In our diabetic rats, AITC did not change diabetic renal status. Interestingly, the highest AITC dose (25 mg/kg b.w.) revealed explicit harmful effects on pancreas as well as on thyroid gland (hypertrophy). It also aggravated the homeostasis model assessment HOMA indices resulted in robust ketosis. Another interesting AITC effect was observed on the body weight gains. Only in diabetic rats administered AITC 5 mg considerably preserved the body weight gains. However, AITC given at the highest dose showed a body weight-reducing effect in diabetic rats. Previously, growth retardation caused by AITC was observed only at considerably high doses and/or after long-term treatment to healthy rats. Final body weight was decreased in animals given high doses of AITC (100 and 150 mg/kg) for 3 consecutive days [25,26]. Another study lasting 103 weeks with daily administration of 12 or 25 mg/kg AITC to healthy rats caused a slight, dose-related decrease in body weight gain [27]. Surprisingly, these all data, including ours, are not consistent with very recent research conducted by Sahin et al. [15]. The authors did not detect any side effect by oral administration of 100 mg/kg b.w. AITC for 12 weeks to diabetic Wistar rats. Based on pharmacokinetics profiles, the maximum concentration of blood AITC metabolites was at the level up to 140 µM after oral administration of 25 mg/kg b.w. AITC [16]. Moreover, such concentration was proved as the upper level of non-cytotoxic concentrations of the AITC in numerous in vitro experiments [16,28,29,30,31]. In vivo study, AITC was demonstrated to possess acute toxicity at the dose LD_50_ 490 mg/kg b.w. for healthy male rats of an unspecified strain [32]. The dose of AITC for its chronic toxicity and pathologic effects was set at 25 mg/kg [31] for 13-week study. In the present study, AITC was used at 2.5; 5 and 25 mg/kg b.w., the doses commonly used in many aspects of research [6,14,16,24,29,33]. Such oral doses achieve reasonable non-toxic concentrations in blood serum in healthy rodents [16]. However, conversely to effects induced in healthy animals, our results indicate that AITC administered to diabetic rats induces plentiful toxic effects.

Diabetes is a group of metabolic disorders that is characterized mainly by elevated levels of glucose in blood (hyperglycemia) and insufficiency in production or action of insulin. In our study, the only beneficial effect of AITC pertains to glycaemia improvement in high fat-diet/streptozocin-induced diabetic rats. The first mention about blood glucose lowering effects of AITC was made by Muztar et al. [34]. A highly considerable decrease in the plasma glucose levels was noted collaterally with uric acid and creatinine drop in male Spraque-Dawley rats fed diets containing 0.1% and 0.3% AITC for 30 days. AITC was recognized by the authors as a potent diuretic agent, which elevated 24 h glucose, creatinine and uric acid excretion with urine. In our study, decrease in blood glucose levels was shown in rats treated with 2.5 and 5 mg of AITC, however, reduced creatinine concentration in the blood serum was observed only at AITC 5 mg/kg b.w. This suggests that there must have been another cause of anti-hyperglycemic action of AITC than elevated urine excretion. Recently, it was proved that AITC significantly improved glucose tolerance by inducing glucose transporter 4 (GLUT4) translocation. Ahn et al. [13] showed that AITC increased glucose uptake in insulin-resistant C2C12 myotubes and augmented GLUT4 translocation in L6-GLUT4myc cells. In our study, skeletal muscle glycogen was without changes at a simultaneous glucose and insulin drop in blood serum. According to Mori et al. [35], AITC affects thermogenesis and energy metabolism via transient receptor potential channel vanilloid type 1 (TRPV1) increasing the utilization of exogenously administered glucose by its oxidation. The dose of 25 mg/kg b.w. was considered to be sufficient to affect energy metabolism. This suggests that improvement in glucose transporter GLUT4 could be equally linked to TRPV1 activation by AITC in diabetic conditions. AITC doses below 25 mg/kg b.w. seemed to achieve this effect. In diabetic insulin resistance, when glucose transport to hepatocytes is limited, the gluconeogenesis process is activated. This process correlates with an increase in alanine aminotransferase activity (ALAT). ALAT is a key enzyme for gluconeogenesis. ALAT catalyzes the transfer of an amino group from alanine to alpha-ketoglutarate pyruvate as a part of this process. In our study, the serum glucose concentration decrease was accompanied by a substantial decrease in alanine aminotransferase activity level at all used AITC doses. This drop seemed to be the response to glucose improvement transport into cells by AITC in diabetic conditions. The ability of down-regulate the gene and protein expression of gluconeogenetic enzymes [Glucose-6 phosphatase (G6P), phosphoenolpyruvate carboxykinase (PEPCK) and glucokinase (GcK)] by isothiocyanates in healthy and diabetic conditions has just been proved [13,36,37]. Very recently, Sahin and co-researchers [15] indicated that 100 mg/kg b.w. AITC decreased the blood glucose concentrations by the improvement in GLUT2 expression in liver and kidney of diabetic rats. In these conditions, also the ketosis process is limited. Such substantial drop in beta-hydroxybutyric acid level was noted at two AITC lower doses used in our experiment.

Our study has shown that treatment of diabetic rats with 5 mg of AITC was associated with a significant decrease in blood insulin levels. This clearly indicates that such dose of AITC worsened the endocrine functions of the insulin-secreting cells in diabetic rats. This negative effect may result from the direct effects of AITC on the insulin-secreting cells. Results of other studies indicate that AITC may induce endoplasmic reticulum stress and mitochondrial damage, which is associated with depletion of intracellular energy. Moreover, AITC may markedly increase formation of reactive oxygen species (ROS) [38]. Glucose-induced insulin secretion is strongly dependent on mitochondrial metabolism and ATP formation [39]. It is also known that pancreatic β-cells are very vulnerable to the ROS-mediated oxidative damage, because of low activity of antioxidative defense mechanisms, compared with other kinds of cells [40]. This strongly suggests that AITC damages β-cells in diabetic rats, via the above-mentioned changes, and thereby reduces blood insulin levels. However, a decrease in insulinemia was accompanied by a simultaneous decrease in blood glucose levels. This suggests that AITC markedly improves insulin action. This assumption is confirmed by results showing that AITC reduced HOMA-IR index. Our results are in agreement with previous observations of Ahn et al. [13]. They reported that AITC improved insulin sensitivity by enhanced mitochondrial enzyme activity in skeletal muscle and liver as a result of increased mitochondrial membrane potential and mitochondrial DNA content. Despite its ability to mitochondrial activity improvements, in our trial, only AITC at dose 5 mg/kg b.w. effectively lowered insulin resistance, as evidenced by reduced HOMA-IR index. The beneficial effects of AITC given at the dose of 5 mg on insulin resistance and on blood glucose levels seems to be partially associated with decrease in blood glucagon. This hormone is well known to exert hyperglycemic effects. The principle of lowering glucagon in the pathophysiology of T2DM seems to be the superior strategy for therapeutic intervention [41], which seemed to be achieved by 5 mg/kg b.w. AITC.

Collaterally to glucose, the key physiological regulators of insulin secretion are incretin hormones, such as glucose-dependent insulinotropic polypeptide (GIP) and glucagon-like peptide-1 (GLP-1). These hormones show much promise in rodent models to decrease blood glucose [42]. Mainly GIP has insulin-releasing and thereby glucose-lowering properties. Our results showed, that AITC was not able to normalize incretin hormone concentration in diabetic rats. Moreover, blood levels of GLP-1 were additionally reduced in rats treated with the highest dose of AITC. This effect is adverse, because incretins are involved in the regulation of insulin secretion and action. It is known that reduced blood incretin levels markedly contribute to pancreatic β-cell failure and to insulin resistance, which develop in type 2 diabetes.

## 4. Conclusions

AITC showed unfavorable influence on peripheral organs. Irrespectively of the applied dose, AITC deteriorated liver, pancreas, thyroid gland status, which was associated with substantial lipid and hormonal metabolism dysregulation in diabetic rats. Furthermore, AITC at the amount of 25 mg/kg b.w. appeared to be a threshold toxic dose. Our results indicate that AITC may be excluded as a candidate for anti-diabetic agent. Interestingly, despite the plentiful adverse effect, AITC at lower doses (2.5 and 5 mg) improved glycaemia and insulin action. Further studies on glucose metabolism (liver gluconeogenesis and lipogenesis) and insulin sensitivity are needed to clarify this hypoglycemic potential of AITC.

## 5. Materials and Methods

### 5.1. Induction of Type 2 Diabetes (T2DM)

The initial weight of rats was 180–200 g (8 weeks old). Rats were purchased from Breeding Lab Animals (Brwinow, Poland). They were maintained under a standard dark-light cycle (12:12 h) at a room temperature of 21 ± 2 °C and humidity (55 ± 5%). Initially, rats were divided into two groups: control (C) (*n* = 8) and diabetic (T2DM) (*n* = 42). The control rats were fed a standard laboratory diet for rodents (Labofeed B, Kcynia, Poland), whereas rats from T2DM group received a high-fat diet (HFD; rodent diet with 40% kcal fat source; Labofeed B) and water ad libitum. After six weeks, rats fed an HFD were intraperitoneally injected with streptozotocin (STZ; 35 mg/kg b.w. dissolved in citric buffer, pH 4.4; 10 mL/kg b.w.). Rats from the control group were injected only with the citric buffer. Animals were further fed the same diets until the end of the experiment. The presence of diabetes was confirmed 3 days after the injection of STZ by glucose tolerance test (GTT) and insulin tolerance test (ITT). Rats treated with STZ with blood glucose levels above 14 mM (≥250 mg/dL) were considered as diabetic and were used in the study.

### 5.2. AITC Administration

To determine the effects of AITC in diabetic rats, the animals were divided into five groups of eight rats each–one control and four diabetic. Rats from the control group and one group of diabetic rats were given deionized water, whereas the remaining diabetic rats were treated with water solution of AITC at the dose of 2.5, 5 or 25 mg per kg b.w. Water or AITC solution (0.5 mL/100 g b.w) were given intragastrically once a day for 14 days. AITC was employed always as a freshly prepared solutions, and body weight was determined every second day.

After two weeks, the animals were slaughtered after overnight fasting (12 h) by decapitation. Blood samples were obtained by exsanguination. The blood serum was taken, and also the livers and skeletal muscles were excised rapidly and kept frozen (−80 °C) until the analysis. Moreover, liver and thyroid mass was determined immediately after decapitation.

### 5.3. Analytical Methods

Levels of hormones were determined in the blood serum using commercial assay kits. Glucose-dependent insulinotropic peptide (GIP) and glucagon-like peptide-1 (GLP-1) were determined using the enzyme-linked immunosorbent assay (ELISA; Phoenix Pharmaceuticals Inc., Burlingame, CA, USA), insulin and glucagon using the radioimmunoassay (RIA) kits specific for rat hormones (Linco Research, St. Charles, MO, USA). Moreover, total T3 and T4, free T3 (fT3) and free T4 (fT4) were measured in blood serum by RIA (DRGInternational, Inc., Springfield, NJ, USA). Liver cholesterol and triglycerides were determined using the enzymatic kits from Pointe Scientific Inc. (Lincoln Park, MI, USA) after extraction of total lipids [43]. The amount of liver and muscle glycogen was determined as blood glucose after extraction in 30% KOH and hydrolysis with amyloglucosidase (12 U/mL; 6000 U/g; SIGMA). Blood levels of total cholesterol, high density lipoprotein cholesterol (HDL-cholesterol), low density lipoprotein cholesterol (LDL-cholesterol), uric acid, creatinine, urea, direct bilirubin, glucose and beta-hydroxybutyric acid were determined using kits from Pointe Scientific Inc. (Lincoln Park, MI, USA). Kits from Pointe Scientific Inc. were also used to determine blood activities of aspartate aminotransferase (AspAT), alanine aminotransferase (AlAT), alkaline phosphatase, pancreatic amylase and pancreatic lipase. Free fatty acids were measured according to Duncombe [44] The De Ritis ratio as diagnostic marker of the liver disease was assayed as AspAT/AlAT. Insulin resistance was characterized by the homeostasis model assessment (HOMA-IR) calculated using the following formula: HOMA-IR = (Fasting gucose[mmol/dm^3^] × Fasting insulin[mUI/dm^3^])/22.5.

### 5.4. Statistical Analysis

Results are presented as the arithmetic mean ± SEM (*n* = 8). The statistical comparison of data was done by one-way ANOVA followed by Tukey’s Multiple Comparison Test at *p* ≤ 0.05 or *p* ≤ 0.01. Only differences were considered between C and T2DM as well as T2DM and T2DM with 2.5; 5 and 25 mg/kg b.w. of AITC, respectively.

## Figures and Tables

**Table 1:** The effect of HFD/STZ injection and AITC administration on carbohydrate and lipid biomarkers in the rat sera, liver and the skeletal muscles.

Parameters	Control	T2DM	T2DM with AITC 2.5 mg/kg b.w.	T2DM with AITC 5 mg/kg b.w.	T2DM with AITC 25 mg/kg b.w.
Serum triacylglycerols (mmol/L)	0.87 ± 0.11	1.88 ± 0.25 **	2.55 ± 0.47 ^†^	2.26 ± 0.90	2.23 ± 0.30
Serum cholesterol (mmol/L)	1.95 ± 0.16	1.61 ± 0.11	2.08 ± 0.36	1.67 ± 0.15	1.82 ± 0.28
HDL cholesterol (mmol/L)	1.12 ± 0.15	1.23 ± 0.16	1.89 ± 0.22 ^†^	1.13 ± 0.06	1.10 ± 0.37
LDL cholesterol (mmol/L)	0.74 ± 0.05	0.49 ± 0.05 **	0.67 ± 0.03 ^††^	0.61 ± 0.08 ^†^	0.60 ± 0.08 ^†^
Free fatty acids (mmol/L)	0.23 ± 0.07	0.17 ± 0.04	0.24 ± 0.04	0.21 ± 0.06	0.28 ± 0.07 ^††^
Liver glycogen (mg/g w.t.)	62.63 ± 7.65	77.31 ± 7.64 *	74.01 ± 7.95	74.76 ± 8.27	70.42 ± 7.85
Muscle glycogen (mg/g w.t.)	6.94 ± 0.91	7.29 ± 1.14	6.76 ± 1.02	7.06 ± 1.49	7.57 ± 1.10
Liver triacylglycerols (mg/g w.t.)	10.59 ± 2.18	11.42 ± 3.11	26.63 ± 7.51 ^††^	22.99 ± 6.07 ^††^	19.11 ± 3.38 ^†^
Liver cholesterol (mg/g w.t.)	2.73 ± 0.13	2.78 ± 0.32	2.46 ± 0.25	2.26 ± 0.27 ^†^	2.59 ± 0.23

Legends: C—control group; T2DM—high-fat diet with STZ injected group; T2DM with AITC 2.5; AITC 5; AITC 25—high-fat diet with STZ injected group with oral administration of 2.5; 5; 25 mg/kg b.w. AITC, respectively. Data are the mean ± SEM (*n* = 8); * *p* ≤ 0.05, ** *p* ≤ 0.01 compared with C group; ^†^ *p* ≤ 0.05, ^††^ *p* ≤ 0.01 compared with T2DM group.

**Table 2:** The effect of HFD/STZ injection and AITC administration on thyroid, pancreas and incretin hormones.

Parameters	Normal Control	HFD/STZ	T2DM with AITC 2.5 mg/kg b.w.	T2DM with AITC 5 mg/kg b.w.	T2DM with AITC 25 mg/kg b.w.
Glucagon (pg/mL)	177.9 ± 14.70	173.7 ± 17.19	157.7 ± 6.67	145.1 ± 11.95 ^††^	157.7 ± 6.48
T4 (nmol/L)	121.1 ± 24.91	113.2 ± 11.50	101.3 ± 25.08	105.8 ±15.42	96.08 ± 28.00
fT4 (pmol/L)	19.93 ± 1.68	13.93 ± 0.57 **	13.25 ± 1.75	11.29 ± 1.61 ^††^	14.08 ± 1.0
T3 (nmol/L)	1.66 ± 0.52	1.72 ± 0.41	1.58 ± 0.32	1.64 ± 0.40	1.66 ± 0.44
fT3 (pmol/L)	4.65 ± 0.57	4.18 ± 0.35	3.53 ± 0.37 ^†^	3.71 ± 0.07	3.31 ± 0.89 ^††^
GIP (mIU/L)	27.22 ± 5.35	18.66 ± 1.24 *	22.71 ± 3.14	22.23 ± 1.19	21.30 ± 6.10
GLP-1 (ng/mL)	0.72 ± 0.15	0.70 ± 0.16	0.51 ± 0.15	0.46 ± 0.15	0.19 ± 0.05 ^††^

Legends: C—control group; T2DM—high-fat diet with STZ injected group; T2DM with AITC 2.5; AITC 5; AITC 25—high-fat diet with STZ injected group with oral administration of 2.5; 5; 25 mg/kg b. w. AITC, respectively. Data are the mean ± SEM (*n* = 8); * *p* ≤ 0.05, ** *p* ≤ 0.01 compared with C group; ^†^ *p* ≤ 0.05, ^††^ *p* ≤ 0.01 compared with T2DM group.

**Table 3:** The effect of HFD/STZ injection and AITC administration on toxicity biomarkers.

Parameters	Control	T2DM	T2DM with AITC 2.5 mg/kg b.w.	T2DM with AITC 5 mg/kg b.w.	T2DM with AITC 25 mg/kg b.w.
Beta-hydroxybutyric acids (mmol/L)	0.52 ± 0.16	3.34 ± 0.34 **	2. 06 ± 0.43 ^††^	1.39 ± 0.23 ^††^	3.07 ± 0.57 ^†^
ALT (IU/L)	20.86 ± 4.55	27.58 ± 4.13 *	19.89 ± 1.53 ^†^	18.12 ± 2.10 ^†^	17.02 ± 4.29 ^†^
AST (IU/L)	63.65 ± 8.42	45.79 ± 7.08 *	43.98 ± 0.85	40.00 ± 5.56	51.63 ± 7.30
De Ritis ratio (AST/ALT)	3.12 ± 0.72	1.68 ± 0.17 **	2.48 ± 0.26 ^††^	2.23 ± 0.24 ^†^	2.7 ± 0.17 ^††^
Direct bilirubin (µmol/L)	0.78 ± 0.25	2.85 ± 0.43 **	4.80 ± 0.36 ^††^	6.200 ± 0.19 ^††^	10.24 ± 1.69 ^††^
Alkaline phosphatase (U/L)	39.78 ± 16.22	235.1 ± 61.67 **	282.9 ± 64.64	287.9 ± 27.58	153.8 ± 55.54 ^†^
Urea (mmol/L)	7.12 ± 0.69	4.78 ± 0.92 *	4.45 ± 0.56	4.74 ± 1.32	5.42 ± 0.30
Creatinine (µmol/L)	58.09 ± 7.47	44.20 ± 6.25 **	42.43 ± 6.85	34.02 ± 1.55 ^†^	44.20 ± 11.57
Uric acid (mmol/L)	1.87 ± 0.22	1.88 ± 0.11	1.82 ± 0.26	1.76 ± 0.09	2.06 ± 0.30
Pancreatic amylase (U/L)	410.2 ± 61.65	410.0 ± 77.92	392.2 ± 99.81	338.9 ± 63.46 ^†^	307.1 ± 51.54 ^††^
Pancreatic lipase (U/L)	20.12 ± 8.73	22.85 ± 7.09	20.80 ± 6.08	18.33 ± 8.40	6.82 ± 3.12 ^†^
Thyroid gland mass (mg/100 g b.w.)	2.78 ± 0.52	2.67 ± 0.20	3.30 ± 0.64	3.53 ± 0.23	3.65 ± 0.25 ^†^
Liver mass (g/100 g b.w.)	2.71 ± 0.26	2.94 ± 0.23	2.96 ± 0.20	2.96 ± 0.17	3.08 ± 0.29

Legends: C—control group; T2DM—high-fat diet with STZ injected group; T2DM with AITC 2.5; AITC 5; AITC 25—high-fat diet with STZ injected group with oral administration of 2.5; 5; 25 mg/kg b. w. AITC, respectively. Data are the mean ± SEM (*n* = 8); * *p* ≤ 0.05, ** *p* ≤ 0.01 compared with C group; ^†^ *p* ≤ 0.05, ^††^ *p* ≤ 0.01 compared with T2DM group.

## Data Availability

Raw data are available from the corresponding author upon request.
